# Synthesis, Properties and Applications of Intermetallics, Ceramic and Cermet Coatings

**DOI:** 10.3390/ma15238408

**Published:** 2022-11-25

**Authors:** Cezary Senderowski

**Affiliations:** Mechanics and Printing Institute, Faculty of Production Engineering, Warsaw University of Technology, Narbutta 85, 02-524 Warsaw, Poland; cezary.senderowski@pw.edu.pl

The production of intermetallic and ceramic protective coatings can be relatively simple, beneficial, and highly predictable. However, comprehensive possibilities for the synthesis and application of this type of coating are still limited by the technological conditions of the synthesis process and coexisting physical phenomena [1]. The physicochemical, thermophysical and structural properties of the coating materials frequently change in situ under synthesis conditions [2,3]. Furthermore, the possible formation of new phases with a nanocrystalline structure in relation to the feedstock powder material could be observed [4,5].

This Special Issue is focused on various conventional synthesis methods of different intermetallic, ceramic, and composite coatings obtained by thermal (D-gun and Arc) spraying [6,7], the CVD process [8,9,10], magnetron sputtering [11], anodization [12], and during the sintering of aluminum, iron and particulate mullite ceramic powders using self-propagated high-temperature synthesis (SHS) [13].

With regard to FeAl-type intermetallic materials, the main challenge is the brittleness of the FeAl (B2) phase due to their long-range ordered (LRO) crystal structure which limits the plastic deformation of the formed material. Therefore, a unique problem involves the analysis of the synthesis conditions’ effect on the deformation and strengthening mechanisms of the nominally brittle intermetallic and ceramic alumina phases formed in situ during the powder particle synthesis processes at the supersonic jet flow in the D-gun spraying (DGS) process [3,6]. This description requires a deep understanding of both the elementary structural transformations of the FeAl powder particles sprayed in the D-gun process into the water and the grain structure in the synthesized intermetallic coating sprayed into the steel substrate [6,14]. On the other hand, the effects of synthesis conditions such as the impact of particle velocity, the temperature, and dynamic pressure of the gaseous stream on the chemical and phase, composition, crystallographic and morphological microtexture, size of the crystallites, and the state of the grain boundaries in the particles and obtained coatings, as well as the degree of superstructure disorder with the identification of nano/ultrafine grain and subgrain areas, dislocation, and antiphase domains, are discussed in detail [6,14].

It should be mentioned that during the thermophysical analysis of both the feedstock powder material and coatings, different phenomena were considered. These include the exchange of momentum and convective heat transfer, as well as the thermal effects of phase changes after the melting of powder particles, and additional analytical and numerical analysis [3,6,14]. It was found in [6] that the gas parameters and thermodynamic state of combustion products at the time of a detonation explosion of the gas mixture in the C–J plane are sufficient to melt FeAl powder particles up to 5 µm in diameter. However, under conditions of thermal forcing in the C–J plane, when a shock wave passes through the powder particles, the changes in particle velocity are much faster than the particle temperature changes caused by convective heating. Therefore, the main factor affecting the heating and melting degree of FeAl powder particles is the gaseous combustion products of detonation.

The thermal diffusivity of Fe40Al at.% powder particles with a diameter of up to 60 µm (mainly determined by the thermophysical properties of the FeAl phase) does not constitute any barrier to reaching the temperature in the particle volume in accordance with temperature changes on its surface. However, the powder particles with a diameter of 40 µm will not be able to absorb enough heat to melt them for the determined thermo-gas-kinetic parameters of the gas-detonation stream and the thermo-physical properties of the Fe40Al at.% phase [6]. In addition, such phenomena are favored by the formation of oxide films on the surface of FeAl powder particles. Such oxide films were found to be formed in the DGS process, during which the passage of the detonation wave front occurs within 10^−7^–10^−5^s, and during the expansion of gaseous detonation products, where partial melting and oxidation on the surface of particles occur.

The layers of Al_2_O_3_ oxides formed naturally in situ under DGS conditions on the surface of FeAl particles additionally constitute a diffusion barrier for heat transfer, typical for oxide ceramics. Thus, it was concluded that the presence of partially melted particles depends also on the heat transfer efficiency resulting mainly from the dynamics of the DGS process as well as the alumina contained in an inhomogeneous composite-like structure formed in situ during the D-gun process [14].

Detailed results presented in [6,14] show that under D-gun spraying conditions, an inhomogeneous composite-like structure of the FeAl coating is formed by unmelted or partially melted particles that underwent geometrical changes during the gas detonation spraying process due to strong plastic deformation.

An alternative way of forming the intermetallic phase-based coating structure from the FeAl system using the arc spraying method was presented by Chmielewski et al. [7]. During the simultaneous melting of two different electrode wires, aluminum and low-alloy steel (98.6 wt.% Fe), a coating structure was obtained in situ in the arc spraying process with the participation of FexAly intermetallic phases. In the second step, high-temperature heating in the range of 700–900 °C for 2 h was applied. During such annealing, the share of FeAl intermetallic phases with a clear ordering of the FeAl (B2) phase after annealing at 900 °C increased, which ensures greater phase homogeneity of the lamellar structure of the coating, with an evident decrease in microhardness [7].

Regarding FeAl-type intermetallics, one long-lasting challenge is to modify these hardly processable materials to improve their consolidation, e.g., by the formation of a ternary matrix also with the participation of dispersion precipitations of oxide ceramics strengthening the matrix. This was possible by using powder mixtures of 28 wt.%. Fe and 52 wt.% Al, also with the participation of 20 wt.% reinforcing mullite ceramics, sintered at high temperature [13]. The theoretical model and further experimental verification proposed by Kopeć et al. [13] enabled the formation of an intermetallic-based composite reinforced with alumina oxides. During the process, the rapid temperature increase, generated during SHS, led to the melting of Al-based metallic liquid. The metallic liquid infiltrated the porous SiO_2_ ceramics, and silicon atoms were transited into the Fe-Al intermetallic matrix. Subsequently, the formation of a Fe–Al–Si ternary matrix and the synthesis of oxygen and aluminum occurred. Synthesis of both these elements resulted in the formation of new, fine Al_2_O_3_ precipitates in the volume of the matrix. A model for the growth of ultra-fine Al_2_O_3_ oxides from the decomposition of SiO_2_ silica during the SHS process was experimentally verified through vacuum sintering, combined with high-energy milling of reinforcement particles. This method enabled us to manufacture an IMC/Al_2_O_3_ composite with a permanent connection between the matrix and reinforcement.

The HfV_2_-HfV_2_O_7_ composite was also proposed as a material with potentially temperature-independent thermophysical properties that could be obtained by combining an anomalously growing thermoelastic HfV_2_ solid with negative thermal expansion HfV_2_O_7_ [11]. Based on the novel design concept, the synthesis of the proposed HfV_2_–HfV_2_O_7_ composite material was performed with two study pathways by (1) the annealing of magnetron-sputtered HfV_2_ thin films in the air to form a HfV_2_O_7_ oxide scale on the thin film surface and (2) the magnetron sputtering of HfV_2_O_7_/HfV_2_ bilayers [11]. Finally, the reduction in the HfV_2_–HfV_2_O_7_ crystalline formation temperature from 550 °C, as obtained upon annealing, to 300 °C using reactive sputtering enables the synthesis of crystalline bilayered HfV_2_–HfV_2_O_7_ to be achieved.

The need for new materials in the photonics industry is reflected in current trends and studies of porous anodic alumina (PAA) as a multifunctional porous ceramic coating prepared by the anodization of aluminum [12]. Intensified research activities on PAA material development for the photonic properties revealed the influence of temperature on the quality of the PAA-based distributed Bragg reflector (DBR) structure fabricated in the oxalic electrolyte in the temperature range of 5–30 °C. This work reported for the first time the production of PAA-based DBR with a good-quality PSB resonance in the mid-infrared (MIR) spectral region, which can extend the application of the PAA-based photonic structures up to the MIR spectral range [12].

Since the extreme performance conditions of modern aircraft engine turbines require the use of heat-resistant materials, MAR 247 nickel-based alloy is also in the scope of the analyzed materials [8,9]. The maximum operating temperature of contemporary nickel superalloys is 1100 °C, which is why it is necessary to use protective coatings on the hot parts of the aircraft engine turbines [15].

The authors of [8] show that the aluminide coatings of various thicknesses and microstructure uniformity deposited by the CVD process performed at different parameters effectively form a thermal barrier and bond coating interacting with the external environment in the air atmosphere at 1100 °C for 24 h under the thermal stability test conditions. The structure and physical-chemical properties, combined with dense and pore-free aluminide coatings obtained by optimized parameters of the CVD process at 1040 °C for 12 h in a protective hydrogen atmosphere, improved the mechanical response, thermal stability, wear resistance and exhibited good adhesion strength to MAR 247 nickel superalloy substrate.

With the increasing demands of the aircraft industry, the increasing application of non-destructive testing to determine the process-dependent properties of the material and to further reduce the amount of experimental work and minimize the manufacturing costs are observed. This is also accompanied by ever-increasing demands for the scientific description of applied measurement methodologies, such as, e.g., “Nondestructive Methodology for identification of local discontinuities in aluminide layer-coated MAR 247 during Its fatigue performance” [9].

In this paper, unconventionally, the fatigue performance of the aluminide layer-coated and as-received MAR 247 nickel superalloy with three different initial microstructures (fine grain, coarse grain and column-structured grain) was monitored using nondestructive, eddy current methods. The aluminide layers of 20 and 40 µm were obtained through the CVD process in the hydrogen protective atmosphere for 8 and 12 h at a temperature of 1040 °C and an internal pressure of 150 mbar. It was found that the elaborated methodology is an effective tool to monitor the degradation of the material. Furthermore, the applied assessment enabled us to localize the area with potential crack initiation and its propagation during 60,000 loading cycles [9]. This was mainly influenced by the initial microstructure of MAR 247 nickel superalloy and the thickness of the aluminide layer synthesized in the CVD process.

As in any field of research, collaboration between different researchers is the key to innovation. Such opportunities are provided by the exchange of experiences based on the research results presented in this Special Issue, “Synthesis, Properties and Applications of Intermetallics, Ceramic and Cermet Coatings”, where publications can be submitted until 30 June 2023.

In this Special Issue, the conditions of synthesis using various methods affecting the structure and functional properties of intermetallic, ceramic and cermet coatings, as well as the analysis of phase transformations, thermophysical properties and other performance properties of produced coatings were studied in detail. Such analysis includes residual stress, adhesion, thermal stability, corrosion resistance and abrasive wear mechanisms, as well as analysis of the geometrical structure of the surface layer of the coatings together with the fractal characterized by using the root mean square (RMS), and other analysis in terms of the comprehensive use of the coatings.

The Editors give special thanks to the authors and the editorial team of *Materials* for the collaborative and peer-review process. We hope you enjoy reading this Special Issue and find new concepts for present and future research works.

## Data Availability

Not applicable.
